# Deciphering the Dilemma: Anticoagulation for Heart Failure With Preserved Ejection Fraction (HFpEF)

**DOI:** 10.7759/cureus.43279

**Published:** 2023-08-10

**Authors:** FNU Jyotsna, Kamran Mahfooz, Haris Sohail, Sumeet Kumar, Maham Adeeb, Dev Anand, Rahul Kumar, FNU Rekha, Giustino Varrassi, Mahima Khatri, Satesh Kumar

**Affiliations:** 1 Medicine, Dr. Bhim Rao Ambedkar Medical College and Hospital, Sahibzada Ajit Singh Nagar, IND; 2 Internal Medicine, Lincoln Medical Center, New York City, USA; 3 Medicine, Lincoln Medical Center, New York City, USA; 4 Internal Medicine, Dow University of Health Sciences, Karachi, PAK; 5 Medicine, Dow University of Health Sciences, Karachi, PAK; 6 Medicine, Bahria University Medical and Dental College, Karachi, USA; 7 Medicine, Liaquat University of Medical and Health Sciences, Karachi, PAK; 8 Pain Medicine, Paolo Procacci Foundation, Rome, ITA; 9 Medicine and Surgery, Dow University of Health Sciences, Karachi, PAK; 10 Medicine and Surgery, Shaheed Mohtarma Benazir Bhutto Medical College, Karachi, PAK

**Keywords:** precision medicine, thromboembolic risk, therapeutic conundrum, heart failure preserved ejection fraction, anticoagulation

## Abstract

Impairment in ventricular relaxation and preserved left ventricular ejection fraction are the two main features of heart failure with preserved ejection fraction (HFpEF) a difficult clinical condition. Therapeutic choices for HFpEF patients are still scarce despite its rising frequency and negative effects on morbidity and mortality, necessitating creative methods to enhance results. The increased thromboembolic risk seen in these individuals raises questions about the relevance of anticoagulation in the therapy of HFpEF. Although anticoagulation has been shown to be beneficial in heart failure with decreased ejection fraction (HFrEF) and other high-risk cardiovascular disorders, its efficacy and safety in HFpEF present a challenging therapeutic challenge. Anticoagulants have been the subject of clinical trials in HFpEF, but the results have been conflicting, giving clinicians only a little information with which to make decisions. The decision-making process is made more difficult by worries about potential bleeding hazards, particularly in susceptible elderly HFpEF patients with other comorbidities. The link between heart failure and anticoagulant medication in HFpEF is thoroughly analyzed in this narrative review. In HFpEF, cardiac fibrosis and endothelial dysfunction create a prothrombotic milieu, as is highlighted in this passage. Also covered are recent developments in innovative biomarker research and cutting-edge imaging techniques, which may provide ways to spot HFpEF patients who might benefit from anticoagulation. This therapeutic conundrum may be resolved by using precision medicine strategies based on risk classification and individualized therapy choices. This review emphasizes the need for more research to establish the best use of anticoagulation in HFpEF within the framework of personalized therapy and shared decision-making. To successfully manage thromboembolic risk and reduce bleeding consequences in HFpEF patients, it is essential to perform well-designed clinical studies and advance our understanding of the pathophysiology of HFpEF. These developments may ultimately improve the prognosis and quality of life for people who suffer from this difficult and mysterious ailment.

## Introduction and background

A crucial clinical entity in cardiovascular medicine, heart failure with preserved ejection fraction (HFpEF), is quickly gaining awareness. Heart failure with preserved ejection fraction (HFpEF), which accounts for around 50% of all cases of heart failure but has historically been overshadowed by heart failure with reduced ejection fraction (HFrEF), has now become a common and difficult condition [1]. Its incidence is anticipated to increase due to an aging population, rising rates of risk factors such as diabetes and hypertension, and improvements in heart failure patients' survival rates [2]. As a result, HFpEF places a significant burden on public health systems and necessitates a full understanding of its pathophysiology as well as the most effective therapeutic techniques.

As their underlying pathophysiological mechanisms differ, it is crucial to distinguish between HFpEF and HFrEF. As a result of poor myocardial contractility, HFrEF has a lower ejection fraction than HFpEF, which is predominantly caused by diastolic dysfunction and a maintained ejection fraction [3,4]. In HFpEF, the heart has trouble relaxing during diastole, which results in insufficient ventricular filling and high ventricular pressures. Due to the combination of increased myocardial stiffness, ventricular-vascular mismatch, and decreased cardiac compliance, HFpEF's characteristic symptoms are exacerbated [5].

Due to the intricacy of HFpEF, there are difficult therapeutic problems that are made even more difficult by the lack of specific, effective pharmacological treatments. Contrary to HFrEF, which has benefited from medicinal treatments based on clinical guidelines such as beta-blockers and angiotensin-converting enzyme inhibitors, HFpEF lacks targeted treatments with proven efficacy [6]. As a result, individuals with HFpEF frequently have limited reactions to standard heart failure drugs, resulting in insufficient symptom alleviation and unfavorable clinical outcomes. The role of anticoagulation has come to light as an exciting topic of study and debate amid the treatment challenges regarding HFpEF. Patients with HFpEF have a higher risk of developing thromboembolic events because of conditions such as atrial fibrillation, endothelial dysfunction, and left atrial stasis [7]. As a result, some scientists and medical professionals have looked at anticoagulation as a possible therapeutic strategy to reduce the risk of stroke and other thromboembolic consequences in HFpEF.

In this narrative review, we seek to present a thorough examination of the complex interaction between HFpEF and anticoagulant medication. We will examine the underlying pathophysiology of HFpEF and the thromboembolic risk factors that are related to it. To further shed light on the possible advantages and safety concerns of anticoagulant usage in HFpEF, we will carefully review the current information from clinical trials and observational research. We'll also look at recent studies on cutting-edge imaging techniques and novel biomarkers that could help us pinpoint which HFpEF patients will benefit from anticoagulation the most. Finally, we will go over the difficulties and limitations of anticoagulant therapy in HFpEF and stress the significance of patient-centered, individualized strategies for managing this therapeutic dilemma.

## Review

Pathophysiology of HFpEF: unraveling the complexity

Diastolic Dysfunction and Myocardial Fibrosis: Major Contributors to HFpEF

Diastolic dysfunction and a preserved left ventricular ejection fraction are the two main features of heart failure with preserved ejection fraction, or HFpEF. Diastolic dysfunction and myocardial fibrosis are two important characteristics that are crucial to the pathogenesis of HFpEF. Clarifying the mechanisms of HFpEF and creating specialized treatment plans require an understanding of how these factors interact. HFpEF-related myocardial fibrosis is the excessive buildup of extracellular matrix elements, such as collagen, in the myocardium is referred to as myocardial fibrosis. It is brought on by an imbalance between collagen synthesis and breakdown, which worsens diastolic relaxation and increases myocardial stiffness. Myocardial fibrosis is a characteristic histological trait seen in the myocardium of people with HFpEF [8]. Myocardial fibrosis in HFpEF develops as a result of numerous variables. A typical characteristic of HFpEF is chronic low-grade inflammation, which is accompanied by the activation of a number of pro-inflammatory cytokines and chemokines. These inflammatory mediators encourage collagen synthesis and fibroblast activation, which results in fibrosis [9]. Furthermore, elevated oxidative stress in HFpEF causes endothelial dysfunction and fibroblast activation, which encourages collagen deposition and fibrosis [10]. By activating profibrotic pathways, neurohormonal imbalances, including elevated levels of angiotensin II and aldosterone, also contribute to the pathophysiology of cardiac fibrosis [11]. Adipokine dysregulation, increased mechanical stress on the heart, and prevalent comorbidities in HFpEF, including hypertension, diabetes, and obesity, all contribute to the growth of myocardial fibrosis through a variety of mechanisms [12]. These related elements foster a profibrotic milieu within the heart, which influences the typical clinical symptoms and diastolic dysfunction seen in HFpEF patients.

Myocardial fibrosis's effects on HFpEF increase myocardial stiffness brought on by myocardial fibrosis and prevent ventricular relaxation during diastole. As a result, the ventricles are unable to fill properly, and left ventricular filling pressures are increased. As a result of the increased stiffness, ventricular-vascular coupling is also impacted, which results in decreased cardiac compliance and diastolic dysfunction [13]. In addition, myocardial fibrosis leads to myocardial remodeling and dysfunction by providing a substrate for arrhythmias. HFpEF diastolic dysfunction is when ventricular relaxation and filling are impaired during diastole; it results in higher filling pressures and decreased ventricular compliance, which is known as diastolic dysfunction. Diastolic dysfunction is the main mechanism causing the clinical heart failure symptoms in HFpEF [7]. In HFpEF, diastolic dysfunction is caused by a number of causes. As was already mentioned, myocardial fibrosis makes the heart more rigid, which affects diastolic relaxation and ventricular compliance. Aspects of poor diastolic function in HFpEF include dysregulated calcium handling and reduced cardiomyocyte relaxation [8]. Myocardial relaxation and diastolic function are further impacted by modifications in the extracellular matrix composition, including an increase in collagen content. Diastolic dysfunction in HFpEF has important clinical ramifications. It results in increased filling pressures during diastole, which causes congestion symptoms such as dyspnea and fluid retention. Patients frequently exhibit pulmonary congestion symptoms, such as enlarged left atrium and high pulmonary capillary wedge pressures. Additionally, atrial fibrillation, a frequent comorbidity in HFpEF, may result from the higher filling pressures, further complicating the clinical course [10]. Diastolic dysfunction is a key component of the pathophysiology of HFpEF and has a significant impact on how symptoms manifest and how patients respond to treatment.

Diastolic dysfunction and myocardial fibrosis are major contributors to the pathogenesis of HFpEF. For the purpose of creating focused therapy strategies to enhance outcomes in HFpEF patients, it is essential to comprehend the mechanisms behind these characteristics. Potential approaches to lessen myocardial fibrosis and relieve diastolic dysfunction in HFpEF include focusing on inflammation, oxidative stress, neurohormonal pathways, and comorbidities.

The Impact of Endothelial Dysfunction and Inflammation on the Progression of Heart Failure with Preserved Ejection Fraction

The progression of HFpEF is significantly influenced by endothelial dysfunction and inflammation. Endothelial dysfunction is characterized by the compromised functionality of the endothelium, the inner lining of blood vessels. The endothelium plays a crucial role in maintaining vascular homeostasis and regulating vascular tone. Endothelial dysfunction plays a significant role in contributing to various pathophysiological processes that worsen the condition of HFpEF.

Nitric oxide deficiency bioavailability: Nitric oxide (NO) is produced by endothelial cells, which act as vasodilators to regulate blood flow and support vascular health. In patients with HFpEF, the availability of nitric oxide (NO) is diminished as a result of reduced activity of endothelial nitric oxide synthase (eNOS) and heightened oxidative stress [14]. This results in compromised vasodilation and heightened peripheral resistance, thereby exacerbating left ventricular filling pressures and contributing to diastolic dysfunction. The presence of chronic low-grade inflammation is a characteristic feature of HFpEF and is linked to the activation of several pro-inflammatory cytokines and chemokines [15]. The inflammatory environment in HFpEF facilitates the activation and impairment of endothelial cells, resulting in heightened leukocyte adhesion to the endothelium, increased permeability, and apoptosis of endothelial cells. The presence of these inflammatory changes contributes to the process of vascular remodeling and microvascular dysfunction, which in turn worsens myocardial stiffness and impairs diastolic function.

Endothelial-cardiomyocyte interactions: The endothelium and cardiomyocytes engage in bidirectional communication via paracrine signaling. Endothelial-derived factors, including endothelin-1 and angiotensin II, have the potential to stimulate profibrotic pathways, thereby playing a role in the development of myocardial fibrosis and diastolic dysfunction [16]. On the other hand, compromised cardiomyocyte function may result in the secretion of substances that exacerbate endothelial dysfunction, thereby perpetuating a detrimental cycle of communication between the endothelium and cardiomyocytes. The presence of endothelial dysfunction and inflammation in HFpEF has been found to have a negative impact on exercise capacity and can contribute to exercise intolerance. The presence of impaired endothelial-dependent vasodilation restricts the blood flow to the muscles engaged in exercise, resulting in premature fatigue and the cessation of physical activity [17]. Additionally, the presence of an inflammatory state in HFpEF has been found to be associated with skeletal muscle abnormalities and mitochondrial dysfunction, which can further hinder exercise performance.

Clinical implications and therapeutic targets: This study explores the clinical implications and potential therapeutic targets associated with the identified phenomenon. The potential therapeutic targets in HFpEF include endothelial dysfunction and inflammation. Strategies focused on enhancing endothelial function and mitigating inflammation may potentially yield favorable outcomes in terms of the progression and symptoms of heart failure with preserved ejection fraction. For example, pharmaceuticals that facilitate vasodilation and improve the availability of nitric oxide, such as angiotensin receptor-neprilysin inhibitors (ARNIs) and phosphodiesterase-5 (PDE-5) inhibitors, have exhibited potential in preliminary research [18]. Furthermore, ongoing research is exploring the potential of anti-inflammatory agents, such as statins and anti-cytokine therapies, to alleviate inflammation and enhance endothelial function in patients with heart failure with preserved ejection fraction. In summary, it can be concluded that endothelial dysfunction and inflammation play a significant role in the pathophysiology and progression of HFpEF. Gaining a comprehensive understanding of the complex relationship between the endothelium, inflammation, and cardiac function is of utmost importance in order to identify innovative therapeutic approaches that can enhance outcomes in patients with heart failure with preserved ejection fraction.

The Interplay between Comorbidities and Risk Factors in the Development of Heart Failure with Preserved Ejection Fraction

The occurrence of HFpEF is influenced by a multifaceted interaction between comorbidities and risk factors. HFpEF frequently manifests in individuals who have multiple concurrent medical conditions. The existence of these comorbidities plays a role in the pathophysiological mechanisms that drive the onset and advancement of HFpEF. Hypertension is a prevalent comorbidity frequently observed in patients with HFpEF. The persistent increase in blood pressure results in the development of left ventricular hypertrophy and fibrosis, which negatively affects the ability of the heart to relax during diastole and increases the stiffness of the myocardium. Consequently, this leads to increased left ventricular filling pressures and diastolic dysfunction, which are prominent characteristics of HFpEF [19]. Diabetes is a notable risk factor associated with the development of heart failure with preserved ejection fraction. Elevated blood sugar levels and reduced sensitivity to insulin contribute to the development of inflammation, oxidative stress, and dysfunction of endothelial cells. These processes are known to contribute to the development of myocardial fibrosis and diastolic dysfunction in HFpEF [20]. The condition of obesity is closely linked to HFpEF. The adipose tissue releases a variety of adipokines and inflammatory cytokines, which contribute to the development of a pro-inflammatory and profibrotic environment. Moreover, heightened adiposity results in mechanical strain on the heart, thereby worsening cardiac remodeling and diastolic dysfunction [21]. AF is a prevalent coexisting condition in HFpEF and is linked to a heightened risk of thromboembolic events. The irregular and rapid contractions of the atria in AF lead to the accumulation of blood in the left atrium, which increases the risk of blood clot formation and subsequent embolic events [22]. Chronic kidney disease (CKD) is commonly observed in patients with HFpEF and is linked to unfavorable clinical outcomes. Decreased renal function results in the retention of sodium and water, which further contributes to fluid overload and exacerbates diastolic dysfunction. CKD also plays a role in the development of systemic inflammation and endothelial dysfunction, which in turn have a significant impact on the pathophysiology of HFpEF [23]. Chronic obstructive pulmonary disease (COPD) is recognized as an autonomous risk factor for the development of HFpEF. Chronic inflammation and elevated pulmonary pressures in individuals with COPD are known to contribute to the development of right ventricular dysfunction and pulmonary vascular remodeling. These changes ultimately have an impact on left ventricular filling and diastolic function [24]. Advancing age and female gender are also considered risk factors for HFpEF. The process of aging is commonly linked to changes in the structure and function of the heart, which include an increase in myocardial fibrosis and a decrease in myocardial compliance. In addition, it is worth noting that female sex hormones have the potential to regulate the cardiovascular response to injury and inflammation, thereby impacting the pathophysiology of HFpEF [25].

Clinical implications and management: In light of the wide range of comorbidities and risk factors associated with the development of HFpEF, it is imperative to adopt a comprehensive and multidisciplinary approach to its management. Improving outcomes for HFpEF can be achieved by implementing proactive measures to address comorbidities such as aggressive blood pressure control, glycemic management in diabetes, weight reduction strategies in obesity, and anticoagulation in AF. In addition, it is imperative to optimize the management of CKD and COPD in patients with HFpEF in order to minimize the impact of these conditions and improve overall quality of life. In summary, the development of HFpEF is influenced by a complex interplay of comorbidities and risk factors. It is crucial to comprehend the correlation between these conditions and HFpEF pathophysiology in order to customize personalized therapeutic approaches and enhance clinical outcomes in patients who are affected.

Thromboembolic risk in HFpEF: evaluating the evidence

The Relationship Between Atrial Fibrillation and Stroke Risk in Heart Failure with Preserved Ejection Fraction

AF and HFpEF are two cardiovascular conditions that frequently occur together and have a synergistic interaction, resulting in heightened morbidity and mortality rates. AF is widely recognized as the most prevalent form of sustained arrhythmia. It poses a substantial risk for stroke, particularly when accompanied by HFpEF. AF is commonly observed in patients with HFpEF, with a reported prevalence ranging from 25% to 50% in various studies [26]. The simultaneous presence of these two conditions is correlated with more unfavorable outcomes in comparison to each condition individually, resulting in an increased likelihood of hospitalizations, exacerbations of heart failure, and occurrences of stroke.

Mechanisms connecting atrial fibrillation and stroke in HFpEF: There are multiple mechanisms that contribute to the elevated risk of stroke observed in patients with HFpEF who also have atrial fibrillation. AF is characterized by irregular and rapid contractions of the atria, leading to the accumulation of blood within the left atrium. The lack of blood flow in this particular area increases the likelihood of thrombus formation, particularly in the left atrial appendage. If these thrombi become dislodged, they have the potential to embolize the cerebral arteries, resulting in a stroke [27]. Both atrial fibrillation and heart failure with preserved ejection fraction are characterized by endothelial dysfunction, leading to an imbalance of vasoactive substances and the promotion of a prothrombotic state. Endothelial dysfunction plays a significant role in promoting platelet activation and adhesion, thereby heightening the likelihood of thrombosis [28]. Both AF and HFpEF exhibit a number of shared risk factors. These include hypertension, diabetes, obesity, and advancing age. These risk factors play a role in the development of both conditions and elevate the overall risk of stroke when they occur together [27]. HFpEF is characterized by compromised vasodilation and abnormal vascular compliance. The vascular alterations described may potentially worsen cerebral small vessel disease, thereby heightening the likelihood of silent cerebral infarcts and microbleeds. These factors, in turn, contribute to an increased risk of stroke [29].

Stroke prevention strategies: It is of utmost importance to implement effective stroke prevention strategies due to the heightened risk of stroke in patients with HFpEF and AF. The primary method for preventing strokes in patients with atrial fibrillation is through the use of oral anticoagulation, which can be achieved with either direct oral anticoagulants (DOACs) or vitamin K antagonists (VKAs). When considering the initiation of anticoagulation, it is important to take into account the CHA2DS2-VASc score. This scoring system evaluates the risk of stroke based on comorbidities and other relevant risk factors [6].

Challenges and considerations: The management of anticoagulation in patients with HFpEF and AF necessitates meticulous consideration, given the intricate balance between stroke prevention and the potential for bleeding, especially in older individuals with multiple comorbidities. The process of making treatment decisions should incorporate shared decision-making, taking into account the patient's individual bleeding risk and personal preferences.

Exploring novel therapies and examining future perspectives: Promising alternative stroke prevention strategies in patients with HFpEF and atrial fibrillation include emerging therapies such as left atrial appendage occlusion devices and non-vitamin K antagonist oral anticoagulants (NOACs). Further research is required to establish the safety and efficacy of these interventions in this particular population [30]. Atrial fibrillation is commonly observed in patients with HFpEF and is associated with a substantial elevation in the risk of stroke. Therefore, it is imperative to prioritize the implementation of strategies aimed at preventing strokes in these individuals. Utilizing a comprehensive methodology for evaluating stroke risk in patients with HFpEF and AF, which includes the CHA2DS2-VASc score, as well as a thorough analysis of individual patient attributes, will greatly enhance the effectiveness of stroke prevention strategies. Future research on innovative therapies and enhanced comprehension of the underlying mechanisms will facilitate the development of more efficacious stroke prevention strategies for this population at high risk.

Non-Atrial Fibrillation Related Thromboembolic Events in Heart Failure with Preserved Ejection Fraction

HFpEF is a clinical syndrome that is distinguished by the presence of heart failure symptoms alongside a preserved left ventricular ejection fraction. While AF is widely recognized as a risk factor for thromboembolic events in patients with HFpEF, it is important to acknowledge that non-AF related thromboembolic events also play a significant role in the morbidity and mortality of this particular group of patients. The purpose of this article is to provide a detailed explanation of two important non-AF-related thromboembolic events in HFpEF and analyze their potential consequences. Arterial thromboembolism is a medical condition characterized by the obstruction of arterial blood flow due to the presence of a blood clot that originates from either the heart or large arteries. Arterial thromboembolism is frequently observed in patients with HFpEF, primarily attributed to the development of intracardiac thrombi. Thrombi frequently form in the left atrial appendage, left ventricle, or both, as a result of blood flow stagnation, endothelial dysfunction, and a prothrombotic state associated with HFpEF [31]. The occurrence of arterial thromboembolism in patients with HFpEF can result in severe outcomes, including stroke, peripheral arterial embolism, and mesenteric ischemia, among various other complications. These events have been found to significantly elevate the risk of mortality and morbidity in patients who are affected [31]. Venous thromboembolism (VTE) is a medical condition that encompasses both deep vein thrombosis (DVT) and pulmonary embolism (PE). Patients with HFpEF are at a heightened risk of VTE due to various factors. These factors include venous stasis, inflammation, endothelial dysfunction, and impaired fibrinolysis [32]. VTE in HFpEF has the potential to worsen symptoms of heart failure, resulting in heightened rates of hospitalizations and diminished quality of life. Furthermore, it is crucial to note that VTE has the potential to result in fatality if it is not promptly diagnosed and effectively managed [32].

Underlying mechanisms: Multiple mechanisms are involved in the heightened risk of non-AF-related thromboembolic events in HFpEF. Chronic inflammation and endothelial dysfunction are frequently observed in patients with HFpEF. These factors contribute to the activation of platelets, the formation of thrombus, and the impairment of fibrinolysis, resulting in the development of a prothrombotic environment [33]. The presence of neurohormonal imbalances in HFpEF, including heightened levels of norepinephrine and angiotensin II, plays a role in the activation and aggregation of platelets, thereby exacerbating the risk of thrombosis [31]. HFpEF is characterized by changes in coagulation factors, such as elevated levels of fibrinogen, factor VIII, and von Willebrand factor. These alterations contribute to an increased risk of thromboembolic events in affected patients [34].

Management: Regarding the management of non-AF-related thromboembolic events in HFpEF, it is imperative to prioritize both primary prevention and the implementation of suitable treatment strategies. In patients with high-risk HFpEF who have a history of thromboembolism, it may be appropriate to consider long-term anticoagulation therapy using medications such as warfarin or DOACs in order to prevent future events [32]. In the case of patients with HFpEF who have experienced arterial thromboembolic events, healthcare professionals may consider prescribing antiplatelet agents such as aspirin or clopidogrel in order to mitigate the likelihood of future occurrences [35]. It is crucial to adopt a proactive approach in managing cardiovascular risk factors, such as hypertension, diabetes, and dyslipidemia, in order to effectively mitigate the overall thrombotic risk in patients with HFpEF [36].

Non-AF-related thromboembolic events play a substantial role in the morbidity and mortality experienced by patients with HFpEF. Gaining a comprehensive understanding of the underlying mechanisms and effectively implementing appropriate prevention and treatment strategies are essential factors in enhancing patient outcomes. Further research in this field has the potential to advance our comprehension and facilitate the development of more efficacious therapeutic interventions.

The Significance of Endothelial Dysfunction in Thromboembolic Complications

Endothelial dysfunction is a multifaceted pathological process characterized by compromised endothelial cell function and diminished bioavailability of nitric oxide (NO). It plays a substantial role in the development of various cardiovascular disorders, such as thromboembolic complications. In the following section, we will examine the significance of endothelial dysfunction in thromboembolic events and its impact on patient management and treatment. The anticoagulant function is impaired. The endothelium plays a pivotal role in the regulation of coagulation through the production of several anticoagulant factors, including tissue factor pathway inhibitor (TFPI) and thrombomodulin. The impairment of endothelial function hinders the production and release of anticoagulant factors, resulting in a state that promotes blood clot formation [37]. The downregulation of TFPI expression, for example, leads to enhanced thrombin generation and subsequent fibrin formation, thereby contributing to the development of thromboembolic complications [38].

Platelet function modification: Endothelial cells secrete NO, which hinders platelet activation and aggregation. Endothelial dysfunction is characterized by a reduction in the availability of NO, which subsequently leads to an increase in platelet activation and adhesion to the endothelium [38]. This phenomenon promotes the development of thrombi rich in platelets, which have the potential to become dislodged and result in thromboembolic events, such as strokes or pulmonary embolisms [39] - the presence of abnormal fibrinolysis. In a properly functioning endothelium, the synthesis and release of tissue plasminogen activator (tPA) occurs to facilitate fibrinolysis, thereby mitigating the risk of excessive clot formation. Nevertheless, the presence of endothelial dysfunction leads to a decrease in the release of tPA and an increase in the production of plasminogen activator inhibitor-1 (PAI-1), thereby impeding the process of fibrinolysis [40]. The discrepancy in the equilibrium between the formation and dissolution of blood clots can result in the presence of enduring thrombi and contribute to the occurrence of thromboembolic events. The topic of interest pertains to endothelial activation and inflammation. Endothelial dysfunction is commonly associated with chronic inflammation, which is characterized by an elevated expression of adhesion molecules and chemokines [35]. Endothelial cells that have been activated play a crucial role in facilitating the recruitment of leukocytes and platelets to the site of injury, thereby augmenting the thrombotic response [41]. Furthermore, it is worth noting that inflammatory cytokines have the potential to directly initiate a prothrombotic state in endothelial cells, thereby exacerbating the occurrence of thromboembolic complications [23].

Clinical implications: The presence of endothelial dysfunction in thromboembolic complications holds considerable clinical significance. Markers of endothelial dysfunction, such as impaired flow-mediated dilation (FMD) or elevated levels of circulating endothelial microparticles, have been linked to a heightened risk of thromboembolic events [36]. These markers have the potential to serve as predictors of thromboembolic risk in a range of cardiovascular diseases. Targeted therapies are a class of medical treatments that specifically target certain molecules or pathways involved in the development and progression of diseases. Gaining a comprehensive understanding of the role of endothelial dysfunction presents the potential for the development of targeted therapeutic interventions aimed at enhancing endothelial function and mitigating thromboembolic risk. Interventions aimed at improving NO bioavailability, such as angiotensin-converting enzyme (ACE) inhibitors or statins, have demonstrated promising potential in reducing thrombotic events [42]. In the context of personalized treatment strategies, the assessment of endothelial function can be valuable in identifying patients who may derive greater benefits from more intensive anticoagulation or antiplatelet therapy. Furthermore, the implementation of risk factor management strategies and the promotion of a healthy lifestyle can play a significant role in preserving endothelial health and mitigating the risk of thromboembolic complications [43]. Endothelial dysfunction is a crucial factor in the development of thromboembolic complications, as it leads to impaired anticoagulant function, altered platelet function, abnormal fibrinolysis, and inflammation. Gaining a comprehensive understanding of the mechanisms that contribute to endothelial dysfunction is crucial for obtaining valuable insights into the development and effective management of thromboembolic events. Targeted therapies and personalized treatment strategies focused on enhancing endothelial health present promising opportunities for mitigating the impact of thromboembolic complications in diverse cardiovascular conditions.

Anticoagulation therapy in HFpEF: balancing risks and benefits

The Management of Anticoagulation Therapy in Heart Failure with Preserved Ejection Fraction: Striking a Balance Between Risks and Benefits

Current guidelines and recommendations for anticoagulation in HFpEF: Patients diagnosed with HFpEF are known to have a heightened susceptibility to thromboembolic events, such as stroke and systemic embolism. Nevertheless, the most appropriate antithrombotic therapy for HFpEF continues to be a subject of discussion owing to the limited availability of comprehensive clinical trial data that specifically addresses this patient population. Consequently, the current guidelines and recommendations for anticoagulation in HFpEF primarily rely on expert consensus and extrapolation from studies conducted in other cardiovascular conditions. The guidelines on HF management by the American Heart Association (AHA) and the American College of Cardiology (ACC), last revised in 2017, recognize the heightened risk of thromboembolism in patients with HFpEF who also have comorbidities such as AF, hypertension, or diabetes. According to the guidelines, it is recommended to administer oral anticoagulants (OACs) for patients with HFpEF and AF based on their CHA2DS2-VASc score [44]. Nevertheless, the guidelines do not offer explicit recommendations concerning anticoagulation for patients with HFpEF who do not have atrial fibrillation. The guidelines for heart failure management by the European Society of Cardiology (ESC), last revised in 2016, also recognize the thromboembolic risk associated with HFpEF. However, these guidelines do not offer specific recommendations regarding anticoagulation therapy for individuals with HFpEF [45]. The guidelines set forth by the Heart Failure Society of America (HFSA), most recently revised in 2017, do not offer explicit recommendations regarding anticoagulation therapy for patients with HFpEF who do not have atrial fibrillation [46].

Comparing the Efficacy and Safety of Anticoagulants and Antiplatelet Agents

When deciding between anticoagulants and antiplatelet agents for patients with HFpEF and no AF, it is important to thoroughly evaluate the efficacy and safety profiles of each option. Anticoagulants, such as warfarin or DOACs, are designed to specifically target the coagulation cascade and effectively prevent the formation of thrombin. On the other hand, antiplatelet agents, such as aspirin or clopidogrel, are intended to inhibit platelet activation and aggregation. The findings from trials, such as the Warfarin versus Aspirin in Reduced Cardiac Ejection Fraction (WARCEF) study, indicate that warfarin may not yield substantial advantages in terms of mitigating the risk of stroke or mortality in patients with heart failure who do not have atrial fibrillation [47]. Additionally, the administration of warfarin for anticoagulation purposes presents an increased susceptibility to bleeding, thereby prompting concerns regarding its safety. On the other hand, antiplatelet agents have demonstrated potential advantages in patients with HFpEF. An illustrative case is the WATCH trial, which provided evidence that aspirin effectively decreased the risk of all-cause mortality among patients with reduced ejection fraction suffering from heart failure [48]. While there is a scarcity of data specific to HFpEF, the favorable safety profile of antiplatelet agents renders them a desirable choice for patients with a lower thromboembolic risk or a higher risk of bleeding.

Assessment of Bleeding Risk in Patients with Heart Failure with Preserved Ejection Fraction

It is essential to conduct a thorough assessment of bleeding risk prior to initiating antithrombotic therapy in patients with HFpEF. Patients with HFpEF frequently present with comorbidities, including renal dysfunction and frailty, which can significantly elevate the likelihood of experiencing bleeding complications. Numerous bleeding risk scores, including the HAS-BLED score, have been validated in various cardiovascular conditions [49]. It is important to acknowledge that the validation of bleeding risk scores for HFpEF patients may be limited. Hence, it is imperative to conduct a thorough evaluation of the various bleeding risk factors, comorbidities, and frailty indicators in order to make informed decisions regarding the administration of anticoagulation or antiplatelet therapy for patients with HFpEF.

Customizing Anticoagulation Therapy to Suit Individual Patient Profiles

Considering the limited availability of conclusive evidence in HFpEF, it is imperative to personalize the decision-making process regarding the initiation of anticoagulation or antiplatelet therapy. This should be done by taking into account the patient's unique clinical characteristics, thromboembolic risk, and bleeding risk. Anticoagulation with OACs is recommended for patients with HFpEF who also have AF, in accordance with established guidelines for AF [47]. When making a decision between warfarin and DOACs, it is important to take into account various factors, including renal function, drug interactions, and patient preference. In the case of patients with HFpEF who do not have atrial fibrillation, the determination of whether to commence anticoagulation or antiplatelet therapy should be made through a thorough evaluation of their individual risk factors. Individuals who have a heightened risk of thromboembolic events, such as a history of such events or significant comorbidities, may find anticoagulation to be advantageous. On the other hand, individuals with a lower risk of thromboembolic events but a higher risk of bleeding may find antiplatelet therapy to be beneficial [48]. It is imperative to closely monitor the patient's clinical status and ensure strict adherence to the chosen therapy, regardless of its nature. It is important to regularly assess renal function and consider potential drug interactions in order to make appropriate dose adjustments and modifications to therapy as necessary. The most appropriate antithrombotic therapy for patients with HFpEF continues to be uncertain due to the absence of specific clinical trial data. The current guidelines primarily emphasize anticoagulation therapy in patients with HFpEF who also have AF. However, the recommendations for HFpEF patients without AF are not as clear and specific. The selection between anticoagulants and antiplatelet agents should be made after careful evaluation of individual patient profiles, taking into account factors such as thromboembolic and bleeding risks, as well as any existing comorbidities. It is crucial to tailor therapy to suit the individual needs of patients and closely monitor their clinical response in order to optimize outcomes for patients with HFpEF.

Clinical evidence and real-world data on anticoagulation in HFpEF

Conducting a Comprehensive Analysis of Pivotal Clinical Trials and Observational Studies

In the realm of managing HFpEF, numerous significant clinical trials and observational studies have investigated the potential impact of anticoagulation therapy. It is worth noting that the majority of these trials primarily focused on patients diagnosed with HFrEF. Consequently, the available evidence supporting the use of anticoagulation in HFpEF is still limited, and it is important to exercise caution when applying findings from trials conducted on HFrEF to this context. The Warfarin and Antiplatelet Therapy in Chronic Heart Failure (WATCH) trial is a prominent study that examined the use of anticoagulation in patients with HFpEF. During the course of this clinical trial, patients diagnosed with heart failure, including individuals with preserved and reduced ejection fraction, were randomly assigned to receive either warfarin, aspirin, or a combination of both medications. The study revealed that there was no statistically significant disparity in the primary outcome of all-cause mortality among the various treatment groups. Nevertheless, subgroup analyses have revealed a potential advantage of aspirin in patients with HFpEF [49]. Another noteworthy study is the Atrial Fibrillation Clopidogrel Trial with Irbesartan for the Prevention of Vascular Events (ACTIVE) trial, which primarily targeted patients diagnosed with AF. During the course of this clinical trial, individuals diagnosed with both AF and heart failure were randomly assigned to one of two treatment groups. The first group received a combination of aspirin and clopidogrel, while the second group received warfarin. Although there was no significant difference in the primary outcome of stroke, systemic embolism, or myocardial infarction between the two groups, there was a noticeable inclination towards decreased occurrences of bleeding events in the aspirin plus clopidogrel group. This suggests that antiplatelet therapy may have a potential role in patients with heart failure [50]. Nevertheless, it is imperative to exercise prudence when interpreting these findings, given that these trials were not explicitly intended to investigate the impact of anticoagulation on HFpEF. Hence, it is imperative to conduct additional research that specifically targets patients with HFpEF in order to obtain more substantial evidence.

Meta-analyses and Systematic Reviews: A Comprehensive Analysis of the Impact of Anticoagulation

In order to obtain additional knowledge regarding the effects of anticoagulation in the management of HFpEF, researchers have conducted meta-analyses and systematic reviews to consolidate data from various studies. A comprehensive meta-analysis was conducted to analyze data from multiple trials and observational studies regarding the utilization of anticoagulation in patients with heart failure, both with and without atrial fibrillation. The analysis indicated that anticoagulation therapy was linked to a reduced risk of stroke, but it also carried a higher risk of major bleeding. This underscores the importance of adopting a personalized approach that takes into account individual risk factors [51]. In a similar vein, a comprehensive review was conducted to examine the effectiveness and safety of antithrombotic treatments, such as anticoagulants and antiplatelet agents, in patients with heart failure, both with and without atrial fibrillation. The analysis revealed that the use of anticoagulation was correlated with a decreased likelihood of experiencing a stroke. However, it is important to note that this benefit was accompanied by an elevated risk of encountering bleeding events. The authors have placed significant emphasis on the importance of tailoring therapy to individual patients, taking into account their unique characteristics and risk profiles [52]. It is crucial to recognize that the evidence derived from these meta-analyses and systematic reviews is still subject to the limitations inherent in the original studies. These limitations frequently encompass diverse patient populations, variations in study designs, and endpoints.

Practical Application of Anticoagulation in the Management of Heart Failure with Preserved Ejection Fraction 

Real-world data offer valuable insights into the practical utilization and outcomes of anticoagulation in patients with HFpEF within routine clinical practice. Research conducted using real-world data has revealed diverse trends in the utilization of anticoagulation therapy among patients with HFpEF. These patterns are influenced by several factors, including the presence of AF, the risk of stroke, and the risk of bleeding. The study employed data from a comprehensive administrative database to evaluate the utilization patterns of anticoagulation in patients with heart failure, both with and without atrial fibrillation. According to the study, patients with HF who also had AF were more inclined to be prescribed anticoagulation therapy in comparison to those without AF. This observation underscores the impact of AF on the decision-making process regarding treatment options [53]. A separate empirical investigation was conducted to assess the results of patients diagnosed with heart failure and atrial fibrillation who were undergoing anticoagulation therapy. According to the study, it was observed that heart failure patients with atrial fibrillation who were undergoing anticoagulation treatment had a reduced likelihood of experiencing a stroke. However, it was also noted that these patients faced an increased risk of major bleeding compared to those who were not receiving anticoagulation therapy [54]. The aforementioned findings highlight the significance of thoroughly evaluating the potential risks and benefits associated with anticoagulation in this specific group of patients. Real-world data also present opportunities for evaluating adherence to anticoagulation therapy and its influence on clinical outcomes. A comprehensive study was conducted to examine the level of adherence to warfarin therapy among patients with heart failure and atrial fibrillation and to determine its potential impact on the risk of stroke. The research study revealed that there is a correlation between inadequate adherence to warfarin medication and a higher likelihood of experiencing a stroke. This highlights the importance of consistently following prescribed medication regimens in order to achieve the best possible outcomes [55]. The available evidence supporting the use of anticoagulation in HFpEF is currently limited. Often, conclusions are drawn from studies conducted in patients with HFrEF or HF patients with AF. Meta-analyses and systematic reviews offer valuable insights into the potential impact of anticoagulation. However, they also emphasize the importance of personalized treatment that takes into account individual patient characteristics and risk profiles. Real-world data provide valuable insights into the practical utilization and outcomes of anticoagulation in routine clinical practice. In order to enhance the existing evidence on the use of anticoagulation in the management of HFpEF, it is necessary to conduct additional research that is specifically tailored to this particular patient population.

Biomarkers and imaging modalities: paving the way for precision medicine

Novel Biomarkers for Thromboembolic Risk Prediction in Heart Failure with Preserved Ejection Fraction

Thromboembolic events, such as stroke and systemic embolism, are notable complications observed in patients diagnosed with HFpEF. The identification of novel biomarkers that can effectively predict thromboembolic risk in this specific population is currently a subject of ongoing research. A number of biomarkers have demonstrated potential in this context:

The D-dimer is a substance that is produced during the breakdown of fibrin, a protein involved in blood clotting. It serves as an indicator of the ongoing process of fibrinolysis. An association has been observed between elevated D-dimer levels and an increased risk of thromboembolic events in patients with HFpEF [56]. The observed phenomenon indicates a continuous presence of prothrombotic and fibrinolytic activity, suggesting its potential utility as a biomarker for predicting the risk of thromboembolism. The Von Willebrand Factor (vWF) is a protein that plays a significant role in hemostasis by facilitating platelet adhesion. Elevated levels of vWF have been associated with endothelial dysfunction and an elevated risk of thrombosis in patients with HFpEF [56]. Soluble P-selectin (sP-selectin) is a cell adhesion molecule that plays a role in platelet activation and leukocyte adhesion. Elevated levels of soluble P-selectin have been found to be correlated with a higher risk of thromboembolic events in patients with HFpEF [56]. N-terminal pro-b-type natriuretic peptide (NT-proBNP) is a widely recognized biomarker used to assess the severity and prognosis of HF. There is a correlation between elevated NT-proBNP levels and an increased risk of thromboembolism in patients with HFpEF [56]. Elevated levels of high-sensitivity troponins have been found to be associated with an increased risk of thromboembolic events in patients with HFpEF. This suggests the presence of myocardial injury and potentially indicates a prothrombotic state [56]. Although these biomarkers exhibit potential in predicting thromboembolic risk, additional research is required to verify their effectiveness through extensive prospective studies.

Exploration of Imaging Techniques for the Identification of High-Risk Patients with Heart Failure with Preserved Ejection Fraction

Imaging techniques are of significant importance in the identification of high-risk HFpEF patients, as they offer valuable insights into cardiac structure, function, and hemodynamics. Several imaging modalities are employed for risk stratification. Echocardiography serves as the principal imaging modality for the diagnosis of HFpEF and evaluation of left ventricular diastolic function. There have been associations found between specific echocardiographic parameters, such as the E/e' ratio (the ratio of early mitral inflow velocity to early diastolic mitral annular velocity), and adverse outcomes as well as an increased risk of thromboembolism in patients with HFpEF [27]. The utilization of cardiac MRI offers comprehensive insights into myocardial structure, fibrosis, and ventricular function. The presence of myocardial fibrosis, as detected by late gadolinium enhancement, has been associated with thromboembolic events in patients with HFpEF [27]. Computed tomography angiography (CTA) is a diagnostic imaging technique that can be utilized to evaluate the presence of coronary artery disease and assess the anatomy of pulmonary veins. These factors are known to be associated with an increased risk of thromboembolism in patients with HFpEF. Nuclear imaging techniques, such as single-photon emission computed tomography (SPECT) or positron emission tomography (PET), have the capability to evaluate myocardial perfusion and metabolism. These techniques offer valuable insights into cardiac function and viability [27].

Incorporation of Biomarkers and Imaging in Clinical Practice: Addressing Challenges and Exploring Opportunities

Incorporating novel biomarkers and advanced imaging techniques into routine clinical practice presents several challenges, despite their potential. Standardization of biomarkers and imaging measurements is imperative across various centers and platforms to guarantee the attainment of consistent and dependable outcomes. Certain biomarkers and imaging techniques may present cost constraints or limited accessibility within various healthcare settings, thereby impeding their widespread utilization. Expertise is necessary for the interpretation of biomarkers and imaging results. The process of integrating this information with other clinical data to make informed treatment decisions can be intricate. Although there is potential in the use of novel biomarkers and imaging techniques for predicting thromboembolic risk in HFpEF, it is crucial to validate their utility through large-scale, prospective studies. Notwithstanding these challenges, the integration of biomarkers and imaging into clinical practice presents substantial prospects for personalized risk assessment and targeted therapy in HFpEF. The early identification of high-risk patients may facilitate prompt interventions aimed at preventing thromboembolic complications and enhancing patient outcomes.

Challenges and limitations in anticoagulation therapy for HFpEF

Insufficient Clinical Trial Data in Heart Failure with Preserved Ejection Fraction Subgroup

HFpEF is a multifaceted and diverse condition, and there has been a scarcity of clinical trial data specifically focused on this subgroup. In the past, the majority of significant randomized clinical trials in heart failure have primarily concentrated on patients with reduced ejection fraction, resulting in a lack of adequate representation of patients with HFpEF in these studies. Establishing evidence-based treatment guidelines for HFpEF has proven to be a challenging endeavor. One of the notable challenges encountered in the execution of clinical trials in HFpEF pertains to the heterogeneity observed within the patient population. HFpEF encompasses a diverse array of etiologies, comorbidities, and clinical presentations, thereby posing challenges in determining a universally applicable therapeutic approach for all patients. Furthermore, the absence of a clearly defined HFpEF phenotype adds further complexity to trial design and the process of selecting patients. Numerous clinical trials have made efforts to incorporate patients with HFpEF. However, these trials have encountered difficulties in attaining sufficient sample sizes, and certain trials have been prematurely discontinued due to futility or the absence of significant treatment effects. The TOPCAT trial, known as the Treatment of Preserved Cardiac Function Heart Failure with an Aldosterone Antagonist, examined the efficacy of spironolactone in patients with HFpEF. The trial yielded neutral findings in relation to the primary outcome, which encompassed cardiovascular death, hospitalization due to heart failure, or aborted cardiac arrest [57]. In recent years, there has been an increasing acknowledgment of the necessity for more focused and individualized strategies in the management of HFpEF. This entails the identification of distinct subgroups within HFpEF, which could potentially exhibit differential responses to therapies and present varying thromboembolic risks. Efforts aimed at fostering collaboration, such as the National Heart, Lung, and Blood Institute (NHLBI) Heart Failure Collaboratory, have been established to advance research in HFpEF and streamline the development of clinical trials for this complex patient population. 

Addressing the Risk of Bleeding in Elderly Patients with Heart Failure with Preserved Ejection Fraction

HFpEF is frequently observed among the elderly demographic. These individuals may exhibit various coexisting medical conditions and are often prescribed multiple medications, factors that can contribute to an elevated susceptibility to bleeding. When evaluating the use of anticoagulation in elderly patients with HFpEF, it is imperative to thoroughly evaluate the delicate equilibrium between the potential advantages of preventing thromboembolic events and the heightened risk of bleeding. Several factors contribute to the risk of bleeding in elderly patients with HFpEF. These factors include age-related alterations in renal function, the presence of other medical conditions such as chronic kidney disease, and the simultaneous use of other medications like nonsteroidal anti-inflammatory drugs. Furthermore, it is imperative to take into account frailty, cognitive impairment, and falls as significant factors that must be considered during the assessment of the appropriateness of anticoagulation in this particular population. To mitigate the risk of bleeding in elderly patients with HFpEF, various strategies can be implemented. The Comprehensive Geriatric Assessment (CGA) is a thorough evaluation conducted to assess the overall health and well-being of older adults. CGA is a comprehensive evaluation that assesses multiple dimensions of an older patient's health, encompassing physical, cognitive, and psychosocial functioning. The integration of CGA into the evaluation process can effectively identify patients who are at a heightened risk of bleeding and provide valuable guidance for treatment decisions. If it is determined that anticoagulation is necessary, optimizing safety can be achieved through dose adjustment that takes into account renal function and comorbidities. The utilization of DOACs is advantageous due to their predictable pharmacokinetics and reduced likelihood of drug interactions when compared to warfarin. Consequently, DOACs may be considered as potentially safer alternatives for elderly patients. Research, such as the ENGAGE AF-TIMI 48 trial, has provided evidence showing that DOACs are not inferior to warfarin in terms of reducing the risk of thromboembolism while also carrying a lower risk of intracranial hemorrhage [58]. It is imperative to conduct regular follow-up visits in order to assess patients' response to anticoagulation therapy and promptly detect any initial indications of bleeding complications. Shared Decision-Making is a collaborative approach in which healthcare professionals and patients work together to make informed decisions about the patient's healthcare. It is imperative to engage the patient in the decision-making process, taking into account their values, preferences, and care objectives, in order to effectively balance the potential risks and benefits of anticoagulation in elderly patients with HFpEF.

Ethical Considerations Play a Significant Role in the Decision-Making Process Regarding Anticoagulation in Patients with Heart Failure with Preserved Ejection Fraction

One of the key ethical considerations involves effectively managing the delicate balance between the risk of thromboembolism and the risk of bleeding. Autonomy and informed consent are crucial in ensuring that patients are provided with comprehensive information regarding the potential risks and benefits associated with anticoagulation. It is essential to incorporate comprehensive information regarding the potential risks of thromboembolic events, bleeding complications, and their respective implications into discussions surrounding informed consent. The principle of beneficence underscores the importance of promoting the well-being of the patient, while the principle of non-maleficence highlights the imperative to prevent harm. The determination to commence anticoagulation therapy should be made through a thorough evaluation of risks, taking into consideration whether the potential advantages outweigh the associated risks. The decision regarding anticoagulation in patients with HFpEF may involve considerations of justice and equity. Ensuring equitable access to treatment options is of utmost importance, taking into consideration various factors, including age, race, and socioeconomic status. In cases of advanced HFpEF, where the emphasis of care may transition towards palliative management, it is important to ensure that decisions regarding anticoagulation are in line with the patient's goals and preferences. The topic of discussion revolves around the concept of shared decision-making and its significance in considering patient values. Shared decision-making is a collaborative process wherein healthcare providers and patients work together to make informed decisions regarding treatment options. It is imperative to have a comprehensive understanding of the patient's values, beliefs, and preferences when making decisions regarding anticoagulation.

The limited availability of comprehensive clinical trial data pertaining to HFpEF, along with the intricate nature of the patient demographic, poses significant obstacles in the process of making anticoagulation decisions. The identification of new biomarkers and the utilization of advanced imaging techniques have the potential to enhance risk stratification in patients with HFpEF. The evaluation and management of bleeding risk, particularly in elderly individuals, necessitates a meticulous assessment and thoughtful consideration of multiple factors. Ethical considerations, such as obtaining informed consent, respecting patient autonomy, and ensuring justice, are integral to the collaborative decision-making process. These considerations are crucial in facilitating personalized and patient-centered care in the management of HFpEF.

Future perspectives: unraveling the conundrum

Ongoing Clinical Trials and Studies: Influencing the Future of Heart Failure with Preserved Ejection Fraction Management

Ongoing clinical trials and studies are currently addressing the knowledge gaps and challenges associated with managing HFpEF. These efforts are actively shaping the future of HFpEF management. The purpose of these trials is to enhance our comprehension of the pathophysiology, ascertain effective treatments, and cater to the specific requirements of patients with HFpEF. One of the notable ongoing trials in HFpEF is the PARAMOUNT-HF trial, also known as the Prospective Comparison of ARNI with ARB on Management of Heart Failure with Preserved Ejection Fraction. The purpose of this trial is to assess the effectiveness and safety of sacubitril/valsartan, an angiotensin receptor neprilysin inhibitor (ARNI), in patients with HFpEF. The findings from the PARAGON-HF trial, also known as the Prospective Comparison of ARNI with ARB Global Outcomes in HFpEF, have demonstrated encouraging results regarding the efficacy of sacubitril/valsartan in reducing the incidence of total hospitalizations for heart failure and cardiovascular mortality among patients with HFpEF [59]. In addition, ongoing clinical studies are investigating potential innovative treatments for HFpEF, including the utilization of sodium-glucose cotransporter-2 (SGLT2) inhibitors and selective cardiac myosin activators. The objective of these studies is to identify potential treatments that can efficiently target the fundamental mechanisms that contribute to the pathophysiology of HFpEF. In addition, current research is examining the potential impact of different biomarkers on the prediction of risk for HFpEF. These biomarkers encompass factors associated with inflammation, fibrosis, and myocardial stress. The findings from these studies have the potential to offer significant insights into the underlying mechanisms of HFpEF and support the advancement of tailored treatment approaches.

Approaches to Personalized Medicine in the Treatment of Heart Failure with Preserved Ejection Fraction

The diverse nature of HFpEF requires the implementation of a personalized medicine strategy in order to maximize patient outcomes. The concept of personalized medicine entails the customization of treatment strategies based on specific patient attributes, such as demographics, comorbidities, genetics, and biomarker profiles. Personalized approaches can offer significant benefits in the management of HFpEF across various aspects. The utilization of innovative biomarkers and imaging techniques can aid in the identification of high-risk HFpEF patients who may derive benefits from targeted treatments or enhanced monitoring. It is imperative to prioritize the management of comorbidities, such as hypertension, diabetes, and obesity when addressing HFpEF. Customizing treatment plans to effectively manage individual comorbidities has the potential to enhance overall outcomes. When making treatment decisions for patients with HFpEF, it is important to take into account various factors, such as the patient's renal function, potential drug interactions, and individual preferences. These considerations can help guide the selection between anticoagulants or antiplatelet agents, whether the patient has atrial fibrillation or not. Achieving optimal diuretic therapy necessitates tailoring the dosage to individual patients, taking into account their renal function and fluid status. The goal is to effectively alleviate congestion without causing hypotension or exacerbating renal function. In specific cases of patients with HFpEF, the utilization of implantable devices such as cardiac resynchronization therapy (CRT) has shown potential to enhance symptoms and mitigate the need for hospitalizations. Promoting personalized lifestyle adjustments, such as dietary modifications, exercise, and weight management, can yield substantial advantages in the management of HFpEF.

Exploring the Potential Role of Novel Anticoagulants in Heart Failure with Preserved Ejection Fraction

Due to the increased susceptibility to thromboembolic events in patients with HFpEF, there is an ongoing investigation into the potential use of novel anticoagulants to mitigate the risk of stroke and systemic embolism in this specific patient group. DOACs have become increasingly appealing due to their predictable pharmacokinetics, minimal drug interactions, and decreased necessity for frequent monitoring in comparison to warfarin. One potential application of DOACs in HFpEF is in patients who also have coexisting AF. AF is a frequently observed comorbidity in patients with HFpEF, and it substantially elevates their susceptibility to thromboembolic events. DOACs, including apixaban, dabigatran, edoxaban, and rivaroxaban, have exhibited non-inferiority or superiority compared to warfarin in the prevention of stroke and systemic embolism among patients with atrial fibrillation [50]. The potential benefits in this particular context also encompass patients with HFpEF who have AF. In addition, DOACs have demonstrated a favorable safety profile, exhibiting a decreased incidence of intracranial bleeding in comparison to warfarin [52]. It is crucial to prioritize this aspect in patients with HFpEF, given the notable concern of bleeding risk, particularly among the elderly or individuals with comorbidities, when deciding on anticoagulant treatment. In light of the considerable potential of DOACs in HFpEF, it is imperative to take into account the unique characteristics of each patient and their susceptibility to bleeding when determining the appropriate course of treatment. Ongoing and future clinical trials are expected to provide further insights into the potential role of DOACs and other anticoagulants in the management of HFpEF. Continuous clinical trials and studies in HFpEF are yielding significant insights into the underlying mechanisms of the condition and informing the advancement of specific therapeutic interventions. The implementation of personalized medicine strategies is essential for optimizing the management of HFpEF. These strategies involve risk stratification, comorbidity management, drug selection, and lifestyle modifications. Novel anticoagulants, specifically DOACs, have the potential to provide advantages in decreasing the risk of thromboembolism in patients with HFpEF, particularly in those who also have atrial fibrillation.

Patient-centric approaches: shared decision-making and adherence

Importance of Patient Education and Engagement in Anticoagulation Therapy

Patient education and engagement are crucial components of anticoagulation therapy in HFpEF patients. Anticoagulation can be a complex and lifelong treatment, and informed and engaged patients are more likely to adhere to therapy, understand potential risks and benefits, and actively participate in shared decision-making. Effective patient education should include the following components. Educating patients about HFpEF, its underlying mechanisms, and the increased risk of thromboembolic events helps them comprehend the importance of anticoagulation therapy. Explaining the purpose of anticoagulation in HFpEF patients, particularly in those with atrial fibrillation, helps patients understand how it reduces the risk of stroke and systemic embolism. Setting clear treatment goals and explaining expected outcomes help manage patient expectations and enhance treatment compliance. Educating patients about the potential risks of bleeding and the benefits of anticoagulation helps them make informed decisions and actively participate in their care. Emphasizing the importance of consistent medication adherence to achieve optimal outcomes is vital, as non-adherence is a significant concern in long-term anticoagulation therapy. Encouraging lifestyle changes, such as a heart-healthy diet, regular exercise, and smoking cessation, can complement anticoagulation therapy and improve overall cardiovascular health. Discussing the need for regular monitoring and follow-up visits helps patients understand the importance of ongoing care and allows healthcare providers to assess treatment efficacy and safety. Engaging patients in their care and decision-making process empowers them to take an active role in managing their health, leading to improved treatment adherence and overall outcomes.

Shared Decision-Making in HFpEF Management

Shared decision-making is a collaborative approach between healthcare providers and patients, involving the exchange of information, exploration of treatment options, and incorporation of patient preferences in clinical decisions. In the context of HFpEF management and anticoagulation therapy, shared decision-making is particularly relevant, considering the complexity of treatment choices and the potential risks and benefits involved. Shared decision-making in HFpEF management involves the following steps:

Information exchange: Healthcare providers should present patients with comprehensive information about HFpEF, thromboembolic risk, and anticoagulation options. This includes discussing the different anticoagulant agents, their mechanisms of action, dosing, potential side effects, and monitoring requirements.

Patient values and preferences: Understanding a patient's values, beliefs, lifestyle, and treatment preferences is crucial in aligning therapeutic choices with individual patient goals. For some patients, avoiding the inconvenience of regular monitoring may lead to a preference for DOACs over warfarin.

Risk-benefit analysis: Healthcare providers should guide patients through a risk-benefit analysis of anticoagulation therapy, considering the patient's individual thromboembolic risk, bleeding risk, and other comorbidities.

Exploring alternatives: In cases where anticoagulation is contraindicated or not preferred by the patient, exploring alternative treatment strategies, such as antiplatelet agents or left atrial appendage closure devices, can be part of the shared decision-making process.

Addressing concerns: Patient concerns, fears, and misconceptions about anticoagulation should be openly addressed to build trust and facilitate shared decision-making.

By involving patients in the decision-making process, healthcare providers can help patients feel more in control of their treatment, improve treatment adherence, and ultimately enhance patient satisfaction and outcomes.

Improving Adherence to Anticoagulation in HFpEF Patients

Adherence to anticoagulation therapy is essential to achieve the desired clinical outcomes, particularly in HFpEF patients at increased risk of thromboembolic events. Improving adherence in this population requires a multifaceted approach:

Patient education: Thorough patient education about the importance of anticoagulation, the potential benefits in reducing thromboembolic risk, and the risks of non-adherence is the foundation of improving adherence.

Tailored treatment plans: Personalizing treatment plans based on individual patient characteristics and preferences can improve treatment acceptance and adherence.

Simplifying regimens: Simplifying medication regimens, such as using once-daily dosing or reducing the number of medications, can improve adherence by reducing complexity.

Reminder systems: Implementing reminder systems, such as smartphone applications or pill organizers, can help patients remember to take their medications as prescribed.

Regular follow-up: Regular follow-up visits with healthcare providers allow for the assessment of adherence, addressing any challenges, and reinforcing the importance of treatment compliance.

Addressing barriers: Identifying and addressing barriers to adherence, such as medication cost, side effects, or forgetfulness, can help patients overcome obstacles to treatment compliance.

Involving caregivers: Involving caregivers or family members in the patient's care can provide additional support and encouragement for adherence.

Digital health solutions: Utilizing digital health solutions, such as telehealth and remote monitoring, can enhance communication between patients and healthcare providers and facilitate better management of anticoagulation therapy.

Health literacy and cultural sensitivity: Ensuring that educational materials are easily understandable and culturally sensitive can improve patients' comprehension and engagement in their care.

Patient education and engagement are integral to the success of anticoagulation therapy in HFpEF patients. Shared decision-making empowers patients to actively participate in their treatment choices, fostering treatment adherence and patient satisfaction. Improving adherence to anticoagulation requires a multifaceted approach that includes personalized education, simplified regimens, reminder systems, regular follow-up, and addressing individual barriers [59]. By integrating these strategies into HFpEF management, healthcare providers can optimize patient outcomes and reduce the risk of thromboembolic events.

## Conclusions

The management of anticoagulation in patients with HFpEF presents a therapeutic challenge due to the intricate nature of the condition and the diverse characteristics of the patient population. However, implementing a multidimensional approach can assist healthcare providers in optimizing anticoagulation therapy and effectively managing HFpEF. By capitalizing on research advancements, well-designed clinical trials that are specific to HFpEF can provide valuable evidence-based guidelines and offer insights into innovative therapies. The integration of personalized medicine, which involves the identification of subgroups within HFpEF and the utilization of biomarkers for risk stratification, enables the customization of treatments for individual patients. The adoption of shared decision-making enables patients to take an active role in their healthcare, ensuring that treatment plans are in line with their preferences and enhancing adherence. Healthcare providers can effectively address therapeutic challenges, improve outcomes, and enhance the well-being of patients in the management of HFpEF by adopting a patient-centered approach. This involves considering the holistic needs of patients and leveraging digital health solutions.
